# Therapeutic potential of natural products and antibiotics against bovine mastitis pathogen of cows and buffaloes

**DOI:** 10.17221/80/2022-VETMED

**Published:** 2023-07-27

**Authors:** Wajid Khan, Shahab Ahmad Khan, Fayaz Akhtar Khan, Sohail Khan, Ikram Ullah, Abdullah Shah, Ihsan Uddin, Sohail Khan, Haji Khan, Muhammad Nazir Uddin, Fazal Akbar, Nabila Qayum

**Affiliations:** ^1^Centre for Biotechnology and Microbiology, University of Swat, Swat, Pakistan; ^2^Veterinary Research and Disease Investigation Center, Balogram, Swat, Pakistan

**Keywords:** antibacterial activity, *Cotoneaster microphyllus*, *mecA* gene, *Pistacia chinensis*, *Staphylococcus aureus*

## Abstract

The present study aims to evaluate the prevalence and antimicrobial sensitivity of *Staphylococcus aureus* associated with bovine mastitis to selected antibiotics and plant extracts. In the current study, 140 milk samples were collected from cows and buffaloes. Among the 140 samples, 93 samples were positive for sub-clinical mastitis based on the California Mastitis Test (CMT). Out of the total positive samples, 45 were confirmed for *S. aureus* on a Mannitol salt agar media. The antimicrobial susceptibility test revealed that 44.82% of the isolates were resistant to cefoxitin (oxacillin) confirming methicillin-resistant *S. aureus* (MRSA) with a higher percentage (51.61%) in the buffalo than in the cow samples. Furthermore, the PCR assay confirmed the presence of the *mecA* gene in all the MRSA isolates. Among the seven tested antibiotics, sulfamethoxazole + trimethoprim showed high efficacy (71.1%) against methicillin-susceptible *S. aureus* isolates (MSSA). Oxytetracycline and sulfamethoxazole + trimethoprim showed 20% efficacy against MRSA followed by enrofloxacin (10%). On the other hand, the tested samples from *Pistacia chinensis* revealed that the ethyl acetate extract of bark showed a maximum zone of inhibition of 21.3 mm against MSSA and MRSA isolates at 3 000 μg/disc. Moreover, the methanol extract of *Cotoneaster microphyllus* formed a 12.3 mm and 9.1 mm zone of inhibition against the MSSA and MRSA isolates, respectively.

Mastitis is an infectious disease among the most prevalent diseases in the livestock sector, causing huge economic losses by affecting the quality and quantity of the milk yield (Halasa et al. 2007). It badly affects the nutritional composition of milk, fat, and caseins (Pyorala 2003). The estimated reduction in the milk yield is 30% per infected quarter and up to a 12% reduction in one of the affected quarters per cow in lactation (Radostits et al. 2007). Among the causative agents of mastitis, *Staphylococcus aureus,* and methicillin-resistant *S. aureus* (MRSA) are the most active and contiguous pathogens (Bergonier et al. 2014). Poor farm management and the extensive use of antibiotics in hospitals and animals have increased the emergence of multi-drug resistant (MDR) *Staphylococcus* MRSA in bovine milk (Indian Network for Surveillance of Antimicrobial Resistance (INSAR) group, India et al. 2013; Resch et al. 2013). Recent research has reported a spa-type MRSA strain CC398 from animals and their farmers known as livestock-associated MRSA (Anjum et al. 2019), showed resistance to various classes of antibiotics, more to tetracycline and lesser to macrolides, lincosamides, aminoglycosides, and fluoroquinolones (Witte et al. 2007). Treatment of an intramammary bacterial infection with antibiotics is complicated due to possible diagnostic errors and lack of therapeutic information (Kvist et al. 2008; Jahanfar et al. 2009).

Efficient treatment policies and proper anti-microbial usage are opposed to the antibiotic resistance in highly pathogenic microbial strains (Wang et al. 2008). Microbial infections have been controlled remarkably with anti-microbial drugs, but the prevalence of resistance of pathogenic bacteria has led to the discovery of different antimicrobial and phytochemical constituents of medicinal extracts, which have the potential to be used for the treatment of microbial infections (Ohno et al. 2003). Medicinal plant extracts have also been used as a treatment for microbial infections and are still providing an alternative treatment for microbial infections (Gurib-Fakim 2006). Additionally, medicinal plant extracts oppose the development of resistance in infectious microorganisms (Ohno et al. 2003). The extracts of different medicinal plants have been reported for anti-MRSA activity (Kumar et al. 2011; Saddiqe et al. 2014; Uysal et al. 2015). Several species of *Cotoneaster* and *Pistacia* have been used in folk medicine for the treatment of several diseases. In different *in vitro* tests, species of the genus *Cotoneaster* have been confirmed for antibacterial, anti-plasmodial, anti-cholinesterase, antioxidant, anti-dyslipidaemic, anti-glucosidase, anti-amylase, and anti-diabetic activities (Esmaeili et al. 2015; Mohamadi Sani and Yaghooti 2016). Similarly, different parts of *Physalis* species have various biological activities like hypoglycaemic, anti-atherogenic antioxidant, anti-inflammatory, and anti-insect activities (Duru et al. 2003; Dedoussis et al. 2004; Ozcelik et al. 2005). As per our knowledge, the different parts of both *C. microphyllus* and *P. chinensis* have not been investigated for their antibacterial potential against the bovine mastitis pathogen.

The emergence of MRSA in bovine milk needs early attention before it goes through the circle from livestock to humans and from humans to livestock and finally develops into severe infectious strains. In the current scenario, proper management and effective therapy by an antimicrobial agent is a prime necessity (Kadlec and Wallace 2009). The present study investigated the prevalence of the *Staphylococcus* species, an *in vitro* therapy and categorised the isolates associated with mastitis in bovine animals by polymerase chain reaction (PCR).

## MATERIAL AND METHODS

### Collection of samples

A total of one hundred and forty milk samples (140) including cows (82) and buffalos (58) were collected from different areas of District Swat, Pakistan in 2018–2019 through professional medical sampling following microbiological techniques. All the milk samples were screened by the California Mastitis Test (CMT) to confirm the range of subclinical mastitis (Dingwell et al. 2003).

### Isolation and identification of *S. aureus* species

*S. aureus* species isolation – CMT-positive milk samples were cultured overnight on a mannitol salt agar media (Oxoid UK, Musaji Adam & Sons, Pakistan) for the *S. aureus* species isolation. The isolates were confirmed by phenotypic and biochemical tests (Table 1), such as Gram staining, tests for oxidation/fermentation, production of phenol, coagulase, catalase, indole-production, methyl red, acetone production, and cefoxitin disc diffusion following Bergey’s manual of systematic bacteriology taxonomy (Collee et al. 1996; Skov et al. 2003).

**Table 1:** Biochemical tests used for the identification of *S. aureus*

Biochemical tests	Results
CMT	positive
Mannitol salt fermentation	positive
Gram staining	positive
Catalase	positive
Coagulase	positive
Indole	positive
Oxidase	negative
Urease	positive
DNase	positive
Acetone production	positive

CMT = California Mastitis Test

### Extraction of DNA and PCR assay

After performing biochemical tests, the DNA was extracted from all 20 isolates using a Genomic ZR Genomic DNATM-Tissue Miniprep kit (Zymo Research Corp, Irvine, CA, USA). All the steps involved in the DNA extraction were performed according to the instructions of the manufacturer. The purity of the isolated DNA was checked by a NanoDrop™ spectrophotometer (2000/2000c) and stored at –20 °C till use. The polymerase chain reaction was undertaken for the amplification of the DNA by targeting the specific gene *mecA* to confirm the MRSA isolates. The PCR reaction mixture included 13 μl of a master mix, 0.5 μl of each primer (forward and reverse), 9 μl of double distilled water, and 2 μl of the DNA sample. The mixture was mixed vigorously and the reaction was performed by a MultiGene Opti Max Thermal Cycler (TC9610) (Labnet International Inc., NJ, USA). The optimised conditions for the PCR reaction were the denaturation step of each cycle at 95 °C for 30 s followed by annealing at 65 °C for 1 min and extension for 1 min at 72 °C (Ahmad and Rahman 2019). Gel electrophoresis was performed for visualising the amplified product on the gel using the Gel doc^TM^ EZ Gel Documentation System (1708270) (BioRad, Hercules, CA, USA).

### *In vitro* susceptibility of the *S. aureus* species to the antibiotics and extracts of medicinal plants

The *in* *vitro* susceptibility of the isolates was checked by seven different antibiotics (Table 2) using a standard Kirby-Bauer disc diffusion test on Mueller Hinton Agar (MHA) media according to the instructions of The Clinical and Laboratory Standards Institute (CLSI) (Wikler 2006). Briefly, the standardised culture (100 μl) was spread evenly over the MHA, and the tested antibiotics were applied. The plates were then kept in an incubator for 24 h at 37 °C. At the end of the experiment, the zone of inhibition was measured in mm (Hudzicki 2009). The same methodology was also applied to test the activity of the medicinal plant extracts against all the isolates.

**Table 2:** Antibiotics, code of the antibiotics, and the disc potency

Serial No.	Antibiotic	Code	Disc potency
1	gentamicin	CN-10	10 μg
2	streptomycin	S-10	5 μg
3	ciprofloxacin	CIP-5	30 μg
4	cefotaxime	CTX-30	30 μg
5	oxytetracycline	OT-30	30 μg
6	enrofloxacin	ENR-5	5 μg
7	sulfamethoxazole + trimethoprim	SXT-25	23.7 μg/1.25 μg

For the preparation of the extracts, the different parts of *C. microphyllus* and *P. chinensis* were dried under shading and then powdered by a grinding machine. The powder plant material (150 g) was then added to the extraction flasks containing one litre of methanol and ethyl acetate. The solutions were then placed in a shaking incubator at 37 °C. These solutions were then filtered through filter paper (Whatman filter paper No. 1; CamLab, Cambridge, UK). The filtrate was then poured into a rotatory evaporator flask and dried via a rotary evaporator at a temperature of 37 °C. The extracts were completely dried through a desiccator. The different concentrations of the extracts were prepared in dimethyl sulfoxide (DMSO) and then tested for anti-bacterial efficacy against methicillin-susceptible *S. aureus* (MSSA) and MRSA isolates using a disc diffusion assay (Khan et al. 2020).

### Statistical analysis

The antibacterial potential of the extracts was expressed as the mean ± standard deviation of the triplicate data of each treatment. The Statistix software (v8.1; Analytical Software, USA) was used for the analysis of data.

## RESULTS AND DISCUSSION

Subclinical mastitis is considered the most significant disease in the livestock industry, causing huge economic losses not only in Pakistan, but also worldwide (Abebe et al. 2016). Bacterial pathogens are evolving day by day leading to the emergence of antibiotic resistance strains, risking public health due to the transmission of resistance to humans and the defectiveness in the antibiotic therapy currently used (El-Sayed et al. 2020). Subclinical mastitis in dairy animals is the source of MRSA bacteria in raw milk samples (Shrestha et al. 2021). In this study, 93 out of 140 (66.6%) bovine animal milk samples were positive for sub-clinical mastitis based on the CMT (Table 3) which is higher than the previous study conducted in Punjab Pakistan (42.2%) and Faisalabad (27%) (Khan and Muhammad 2005; Maalik et al. 2019). The CMT analysis of the milk samples further revealed the prevalence of sub-clinical mastitis was higher in buffalos (75.47%) than in cows (54.05%).

**Table 3:** Prevalence of subclinical mastitis in bovine milk samples assayed by CMT

Bovine animal	Samples tested	CMT +ve	%
Buffalo	82	62	75.60
Cow	58	31	53.44
Total	140	93	66.42

**+**ve = positive; CMT = California Mastitis Test

Furthermore, 45 (48.38%) of 93 samples showed cultural growth on selective media, a higher percentage of MSSA subclinical mastitis was recorded in buffalos (50.0%) than in cows (45.16%) (Table 4). In comparison to our findings, Dieser et al. (2014) reported 53.9%, while a study conducted by Nam et al. (2011) in Korea revealed a 54.3% prevalence of *S. aureus* sub-clinical mastitis in cows, which is near to our findings. The results of the study conducted in South Ethiopia reported a high prevalence rate of *S. aureus* associated with sub-clinical mastitis (74.7%) in the herd (Abebe et al. 2016). Research carried out in various countries showed inconsistencies in evaluating the prevalence rate of mastitis and its pathogenic micro-organisms due to differences in the sample collection, culture, and diagnostic techniques (Dieser et al. 2014). Other predisposing factors are also responsible for the difference in the prevalence rate of subclinical bovine mastitis, including the poor handling of animals and milk, poor sanitation systems, milking with contaminated machines and hands, and poor housing of animals. Moreover, the improper use of antibiotics, severe infections in mammary glands, and the emergence of MDR pathogens are also accountable for the differences in the prevalence rate of bovine mastitis (Seegers et al. 2003). The isolates were further screened for MRSA using a cefoxitin disc diffusion assay. The results of the screening assay revealed 44.44% MRSA among the total isolates (Table 5). A higher percentage (51.61%) of MRSA was noted in the buffalo samples than in the cow samples (28.57%). The high incidence of MRSA in the buffalo samples can be attributed to several risk factors including poor hygienic conditions in their housing system, bad sanitation, and the excessive use of antimicrobial agents for the treatment of mastitis (Algammal et al. 2020; Gwida et al. 2021). The DNA was isolated from the positive samples with the cefoxitin test and subjected to a PCR assay using a specific primer for the *mecA* gene. The results of the experiment revealed that all the isolates of MRSA, already confirmed by the cefoxitin test, have the *mecA* gene in their genomes. A similar finding was also reported by Dibah et al. (2014). The prevalence of MRSA reported in different countries is variable (Guardabassi et al. 2013; Dibah et al. 2014). The current investigation reported a higher incidence of MRSA in the raw milk samples than those in Italy (20%), Turkiye (17%), Germany (2.30%), and England (2.3%) (Turkyilmaz et al. 2010; Kreausukon et al. 2012; Paterson et al. 2013; Riva et al. 2015). The difference in the prevalence rate may be due to differences in the study design, lab testing for identifying MRSA, improper and non-specific use of antibiotics, and other infection control measures (Askari et al. 2012). After categorising the isolates into MSSA and MRSA, the isolates were tested for susceptibility to antibiotics and plant extracts using a disc diffusion assay. The data of the study indicated that a high percentage of MSSA isolates were susceptible to the tested antibiotics, sulfamethoxazole + trimethoprim was found to be the most susceptible antibiotic (Table 6) among all the tested antibiotics (72.2%) followed by gentamicin (66%), streptomycin, and enrofloxacin (each 46.66%). The high percentage of susceptibility of MSSA isolates was also documented against sulfamethoxazole + trimethoprim, gentamicin, and enrofloxacin in previous studies (Kumar et al. 2011; Chandrasekaran et al. 2014; Aqib et al. 2019). Furthermore, the susceptibility of *S. aureus* isolated from different sources showed variations to the different antibiotics (Nam et al. 2011). The high susceptibility of oxytetracycline was reported by Chandrasekaran et al. (2014) which is contrary to our findings. In this study, oxytetracycline was the least susceptible and more intermediate, the decrease in susceptibility of oxytetracycline against *S. aureus* might be due to the acquisition of the day-by-day resistance. Furthermore, comparative antibiotic resistance profiles of *S. aureus* isolated from different food products and clinical specimens against cephems, tetracycline, aminoglycosides, penicillin macrolides, lincosamides, fluoro-quinolones have also been reported in previous studies (Paludi et al. 2011; Jackson et al. 2013; Momtaz et al. 2013; Sallam et al. 2015; Safarpoor Dehkordi et al. 2017).

**Table 4:** *S. aureus* prevalence in bovine milk samples

Specie of animal	Samples tested	CMT +ve	Positive culture	% of* S. aureus*
Buffalo	82	62	31	50.00
Cow	58	31	14	45.16
Total	140	93	45	48.38

**+**ve = positive; CMT = California Mastitis Test

**Table 5:** MRSA prevalence in bovine milk samples by the cefoxitin test and PCR

Animals	Tested samples	% MRSA by cefoxitin test	% MRSA by PCR	% MRSA
Buffalo	31	16	16	51.61
Cow	14	4	4	28.57
Total	45	20	20	44.44

MRSA = methicillin-resistant *S. aureus*; PCR = polymerase chain reaction

**Table 6:** Antibiotics’ zone of inhibition interpretation according to the CLSI with values and the *in vitro* therapeutic efficacy of the antibiotics used against the MSSA isolates

Antimicrobial class	Antimicrobial agents	Concentration	S	I	R	S	I	R
Fluroquinolone	enrofloxacin	5 μg	≥ 21	17–20	≤ 16	21	17	11
Fluroquinolone	ciprofloxacin	5 μg	≥ 21	16–20	≤ 15	19	11	25
Aminoglycoside	gentamicin	5 μg	≥ 15	13–14	≤ 12	30	6	9
Aminoglycoside	streptomycin	5 μg	≥ 15	12–14	≤ 11	21	9	15
Cephalosporin	cefotaxime	5 μg	≥ 23	15–22	≤ 14	19	21	5
Tetracycline	oxytetracycline	5 μg	≥ 26	16–25	≤ 15	10	29	6
Sulfonamide	sulfamethoxazole + trimethoprim	5 μg	≥ 16	11–15	≤ 10	32	2	11

CLSI = Clinical and Laboratory Standards Institute; I = intermediate; MSSA = methicillin-susceptible *S. aureus*; R = resistant; S = sensitive

The current study revealed higher resistance in the MRSA isolates than the MSSA isolates to the most commonly used antibiotics. The highest MRSA resistance was noted for streptomycin, gentamicin, ciprofloxacin, and cefotaxime (90%, 85%, 70%, and 65%, respectively) (Table 7). A similar finding was also reported by Dibah et al. (2014) in which the MRSA isolates were found nearly resistant to all the tested antibiotics. Although oxytetracycline and sulfamethoxazole + trimethoprim showed a minimum of 20% anti-MRSA activity. Our study is contrary to the findings of Chandrasekaran et al. (2014) who reported that most of the MRSA isolates were susceptible to the tested antibiotics. Even though they also reported MRSA as multidrug-resistant in their research study. On the other hand, the extracts from the medicinal plant also showed antibacterial potential against MSSA and MRSA isolates.

**Table 7:** Antibiotics’ zone of inhibition interpretation according to the CLSI with values and the *in vitro* therapeutic efficacy of the antibiotics used against the MRSA isolates

Antimicrobial class	Antimicrobial agents	Concentration	S	I	R	S	I	R
Fluroquinolone	enrofloxacin	5 μg	≥ 21	17–20	≤ 16	2	6	12
Fluroquinolone	ciprofloxacin	5 μg	≥ 21	16–20	≤ 15	1	5	14
Aminoglycoside	gentamicin	5 μg	≥ 15	13–14	≤ 12	1	2	17
Aminoglycoside	streptomycin	5 μg	≥ 15	12–14	≤ 11	0	2	18
Cephalosporin	cefotaxime	5 μg	≥ 23	15–22	≤ 14	1	6	13
Tetracycline	oxytetracycline	5 μg	≥ 26	16–25	≤ 15	4	5	11
Sulfonamide	sulfamethoxazole + trimethoprim	5 μg	≥ 16	11–15	≤ 10	4	6	10

CLSI = Clinical and Laboratory Standards Institute; I = intermediate; MRSA = methicillin-resistant *S. aureus*; R = resistant; S = sensitive

The methanol and ethyl acetate extracts of *P. chinensis* leaves showed a maximum zone of inhibition of 16.68 mm and 15.82 mm against *S. aureus* isolates and 14.58 mm and 12.4 mm zone of inhibition against the MRSA isolates, respectively. The antibacterial efficacy of *P. chinensis* leaves was also reported by Rashed et al. (2016) against different microorganisms. Similarly, the ethyl acetate extracts of the bark of *P. chinensis* revealed high antibacterial activity against these isolates. Ethyl acetate extracts formed a maximum zone of inhibition of 21.3 mm followed by the methanol extract of 14.1 mm against MSSA and a 20.3 mm and 12.7 mm zone of inhibition against MRSA isolates, respectively. The antibacterial potential of methanol extracts of bark against the bacterial species was also reported in the previous research study (Hazrat et al. 2013). Furthermore, methanol and ethyl acetate solvent extracts of the fruit of *P. chinensis* revealed a 15.19 mm and 14.44 mm zone of inhibition against MRSA isolates while a maximum zone of inhibition of 20 mm and 19.2 mm against MSSA isolates was found (Tables 8 and 9). The antimicrobial potential against different gram-positive bacteria has been reported in the fruit of *Pistacia integerrima* (Ahmad et al. 2013).

**Table 8:** Average zone of inhibition of the MRSA isolates produced by the medicinal plant extracts at 1 000, 2 000 and 3 000 μg**/**disc

Plants	Parts used	Extract	Zone of inhibition (mm)		
			1 000 μg/disc	2 000 μg/disc	3 000 μg/disc
*P. chinensis*	fruits	methanol	9.23 ± 1.0	13.35 ± 1.1	15.2 ± 1.4
		ethyl acetate	8.13 ± 0.9	11.93 ± 0.9	14.4 ± 1.3
*P. chinensis*	leaves	methanol	8.69 ± 0.6	11.41 ± 0.9	14.8 ± 1.2
		ethyl acetate	4.17 ± 0.1	10.24 ± 1.0	12.4 ± 1.1
*P. chinensis*	bark	methanol	5.0 ± 0.3	9.93 ± 1.0	12.7 ± 1.2
		ethyl acetate	10.37 ± 1.0	15.44 ± 1.2	21.3 ± 1.6
*C. microphyllus*	leaves	methanol	3.15 ± 0.9	7.28 ± 0.9	9.1 ± 1.1
		ethyl acetate	2.91 ± 0.7	5.31 ± 0.8	6.4 ± 1.0

mm = millimetre; MRSA = methicillin-resistant *S. aureus*

**Table 9:** Average zone of inhibition of the MSSA isolates produced by the medicinal plant extracts at 1 000, 2 000 and 3 000 μg**/**disc

Plant species	Parts used	Extract	Zone of inhibition (mm)		
			1 000 μg/disc	2 000 μg/disc	3 000 μg/disc
*P. chinensis*	fruits	methanol	10.23 ± 1.0	15.35 ± 1.1	20.0 ± 1.4
		ethyl acetate	10.13 ± 0.9	13.93 ± 0.9	19.2 ± 1.3
*P. chinensis*	leaves	methanol	9.79 ± 0.6	13.31 ± 0.9	16.6 ± 1.2
		ethyl acetate	0.7 ± 0.3	11.24 ± 1.0	14.8 ± 1.1
*P. chinensis*	bark	methanol	6.0 ± 0.3	10.93 ± 1.0	12.7 ± 1.2
		ethyl acetate	13.31 ± 1.0	16.34 ± 1.2	21.3 ± 1.6
*C. microphyllus*	leaves	methanol	5.75 ± 0.9	9.58 ± 0.92	12.3 ± 1.1
		ethyl acetate	5.41 ± 0.7	7.51 ± 0.8	9.82 ± 1.0

mm = millimetre; MSSA = methicillin-susceptible *S. aureus*

The methanol and ethyl acetate extracts of the leaves of *C. microphyllus* showed a maximum zone of inhibition of 12.34 mm and 9.82 mm against MSSA isolates while a 9.1 mm and 6.4 mm zone of inhibition against MRSA isolates was seen, respectively (Tables 8 and 9). The antibacterial potential of this genus *C. microphyllus* is also been known for the other species of this genus, which supports the current findings of the study. Furthermore, the antibacterial potential of *Cotoneaster nummularius* against various human pathogenic bacteria is due to the inclusion of phenolic and flavonoid bioactive constituents, as reported in previous studies (Zengin et al. 2014; Uysal et al. 2016). On the other hand, both the methanol and ethyl acetate root extracts of *C. microphyllus* were inactive against both MSSA and MRSA isolates. The inactivity of the root extracts might be due to the uneven distribution of the bioactive compound in the different parts of the plant.

Furthermore, according to Taylor et al. (2001), the absence of the antibacterial potential does not show the absence of bioactive compounds in the roots, but there may be an insufficient amount of bioactive compounds in the solvent extracts to validate the biological activities at the used dosage level (Lindsey et al. 1999).

In conclusion, it could be stated that both MRSA and MSSA isolates causing bovine mastitis were resistant to the commonly used antibiotics. The extracts of the medicinal plants showed promising results in the current study and can be used as an alternative drug for the control of bovine mastitis in cows and buffaloes.
